# Deciphering the mechanism of *Tinospora cordifolia* extract on Th17 cells through in-depth transcriptomic profiling and *in silico* analysis

**DOI:** 10.3389/fphar.2022.1056677

**Published:** 2023-01-09

**Authors:** Amrita Nandan, Vishwas Sharma, Prodyot Banerjee, Kannan Sadasivam, Subramanian Venkatesan, Bhavana Prasher

**Affiliations:** ^1^ Genomics and Molecular Medicine, Council of Scientific and Industrial Research -Institute of Genomics and Integrative Biology (CSIR-IGIB), Delhi, India; ^2^ CSIR’s Ayurgenomics Unit, Translational Research and Innovative Science Through Ayurgenomics (TRISUTRA), CSIR-IGIB, Delhi, India; ^3^ Centre of Excellence for Applied Development of Ayurveda, Prakriti and Genomics, CSIR- IGIB, Delhi, India; ^4^ Independent Researcher, Prayagraj, India; ^5^ Centre for High Computing, CSIR-Central Leather Research Institute (CLRI), Chennai, India; ^6^ Department of Chemistry, Indian Institute of Technology Madras, Chennai, India

**Keywords:** immunomodulatory, ayurveda, guduchi, molecular docking, signaling pathway, Th17 cells, *Tinospora cordifolia*

## Abstract

Naive CD4^+^ T cells differentiate into effector (Th1, Th2, Th17) cells and immunosuppressive (Treg) cells upon antigenic stimulation in the presence of a specific cytokine milieu. The T cell *in vitro* culture system provides a very efficient model to study compounds’ therapeutic activity and mechanism of action. *Tinospora cordifolia* (Willd.) Hook.f. & Thomson (Family. Menispermaceae) is one of the widely used drugs in *Ayurveda* (ancient Indian system of medicine) for various ailments such as inflammatory conditions, autoimmune disorders, and cancer as well as for promoting general health. *In vitro* and *in vivo* studies on immune cells comprising dendritic cells, macrophages, and B cells suggest its immune-modulating abilities. However, to date, the effect of *T. cordifolia* on individual purified and polarized T cell subsets has not been studied. Studying drug effects on T cell subsets is needed to understand their immunomodulatory mechanism and to develop treatments for diseases linked with T cell abnormalities. In this study, we examined the immunomodulatory activity of *T. cordifolia* on primary CD4^+^ T cells, i.e., Th1, Th17, and iTreg cells. An aqueous extract of *T. cordifolia* was non-cytotoxic at concentrations below 1500 µg/ml and moderately inhibited the proliferation of naive CD4^+^ T cells stimulated with anti-CD3ε and anti-CD28 for 96 h. *T. cordifolia* treatment of naive CD4^+^ T cells differentiated under Th17-polarizing conditions exhibited reduced frequency of IL-17 producing cells with inhibition of differentiation and proliferation. For the first time, in-depth genome-wide expression profiling of *T. cordifolia* treated naive CD4^+^ T cells, polarized to Th17 cells, suggests the broad-spectrum activity of *T. cordifolia*. It shows inhibition of the cytokine-receptor signaling pathway, majorly *via* the JAK-STAT signaling pathway, subsequently causing inhibition of Th17 cell differentiation, proliferation, and effector function. Additionally, the molecular docking studies of the 69 metabolites of *T. cordifolia* further substantiate the inhibitory activity of *T. cordifolia via* the cytokine-receptor signaling pathway. Furthermore, *in vitro* polarized Th1 and iTreg cells treated with *T. cordifolia* extract also showed reduced IFN-γ production and FoxP3 expression, respectively. This study provides insight into the plausible mechanism/s of anti-inflammatory activity of *T. cordifolia* involving T cells, mainly effective in Th17-associated autoimmune and inflammatory diseases.

## Introduction

T cells are a crucial component of the adaptive immune system (Claeys and Vermeire, 2019). Many natural plant products are known to modulate immunity, and their effects have been extensively studied on immune cells to understand their immunomodulatory, prophylactic, and therapeutic functions (Jantan et al., 2015). The stem of the *Tinospora cordifolia* (Willd.) Hook.f. & Thomson (Family Menispermaceae) (TC) plant, also known as Guduchi, is widely used in ayurvedic medicine (ancient Indian system of medicine) and other traditional medicine for treating various ailments through the restoration of homeostasis (Upadhyay et al., 2010; Ministry of Ayush, Govt of India, New Delhi, 2022). TC is suggested as an immune booster and is consumed as a crude extract, decoction, or tablet with an adult dose of 500 mg twice daily (Upadhyay et al., 2010). Based on ancient knowledge and several *in vitro* and *in vivo* studies on animal models, TC is shown to have anti-angiogenic, anti-arthritic, osteoprotective, and immunomodulating properties (Prince and Menon, 1999; Leyon and Kuttan, 2004; Raveendran Nair et al., 2004). These properties are due to various biochemicals comprising alkaloids, glycosides, diterpenoid lactones, sesquiterpenoids, phenols, and polysaccharides that influence the cellular function of immune cells (Singh et al., 2003; Sreenivasa Reddy et al., 2009).

The immune response involves several types of cells derived from either 1) myeloid cells: monocytes, macrophages, and dendritic cells, or 2) lymphoid cells: T cells and B cells (Janeway and Medzhitov, 2002). TC activity on macrophages is well established both *in vitro* and *in vivo* (Alrumaihi et al., 2019; Reddi and Tetali, 2019). G1-4A, an acidic arabinogalactan derived from the stem of TC, acts as a non-microbial TLR4 agonist, which leads to macrophage activation, and induces B cell proliferation (Raghu et al., 2009). Immunomodulatory protein (Guduchi ImP) isolated from the stem of TC shows mitogenic activity on murine thymocytes and splenocytes, as well as human peripheral blood lymphocytes (Aranha et al., 2012). Bone marrow-derived dendritic cells acquire a killer phenotype and exhibit tumoricidal activity in the presence of G1-4A (Pandey et al., 2012; Pandey et al., 2014). (1,4)-α-D-glucan (RR1) from TC activates macrophages through NF-κB translocation, TLR6 signaling, and cytokine production (Nair et al., 2006; Upadhyaya et al., 2011). RR1 is also demonstrated *in vitro* to stimulate human lymphocytes, resulting in the synthesis of inflammatory cytokines (Raveendran Nair et al., 2004). TC reverses the SARS-CoV-2 viral spike-protein induced disease phenotype in a humanized zebrafish model system (Balkrishna et al., 2021a). Recent docking studies have shown Tinocordiside, a metabolite of TC, as a viable option for controlling SARS-CoV-2 infection and its entry into host cells (Balkrishna et al., 2021b).

CD4 T cells are fundamental in orchestrating an adaptive immune response. CD4 T cells broadly comprise Th1, Th2, Th17, and Treg (T regulatory) cells based on the expression of signature cytokines and lineage-specific master transcription factors. Th1 cells are characterized based on their interferon-γ (IFN-γ) production. IL**-**12 and IFN-γ concomitantly signal through the transcription factors STAT4, STAT1, and T-bet, to differentiate naive T cells towards the Th1 lineage (Szabo et al., 2000). T-bet is considered the master regulator of Th1 cell differentiation (Zhou et al., 2009). These cells are essential in providing immunity against intracellular pathogens such as viruses and certain bacteria (Mosmann and Coffman, 1989). They are also responsible for the induction of some autoimmune diseases in humans (Zhu and Paul, 2008). Th2 cells are characterized based on the production of IL-4, IL-5, and IL-13 and the expression of the master regulator GATA3. These cells provide immunity against various extracellular pathogens and helminths, and are key players in inducing allergic inflammatory diseases such as asthma to persist (Zhou et al., 2009). The production of the pro-inflammatory cytokines IL-17A and IL-17F identify Th17 cells. The cytokines transforming growth factor-beta (TGFβ), IL-6, and IL-21 and transcription factors RORγt and STAT3 collectively contribute to the differentiation of naive T cells toward Th17 cells (Janeway and Medzhitov, 2002; Dong, 2008). Treg is marked by the production of IL-10, TGFβ, and IL-35, with Foxp3 expression as the master transcription factor. The master regulator Tregs controls Th1, Th2, and Th17 responses to maintain homeostasis and are involved in preventing autoimmune diseases (Zhu and Zhu, 2020).

Th17 cells are crucial in the adaptive immune response. IL-6 and TGFβ are responsible for activating STAT3 *via* the JAK-STAT signaling pathway, followed by the differentiation of naive CD4^+^ T cells to Th17 cells and induction of several Th17-associated genes such as IL-17, Rorc, and IL-23R. In addition, cytokines such as IL-21 act as autocrine factors and promote the differentiation of Th17 cells through a positive feedback mechanism. Similarly, for terminal differentiation and maintenance, Th17 cells require IL-23 (Stritesky et al., 2008). Th17 cells play an important role in providing immunity against predominantly extracellular bacterial and fungal pathogens. They also maintain a barrier function at mucosal sites (Zúñiga et al., 2013). Th17 cells and Th17 cytokines are important for the pathogenesis of several autoimmune and inflammatory diseases, including psoriasis, psoriatic arthritis, multiple sclerosis, rheumatoid arthritis, Crohn’s disease, systemic lupus erythematosus, *etc.* Therefore, inhibiting the IL-17/IL-17R pathway is suggested as a useful method for treating autoimmune inflammatory disorders (Yamagata et al., 2015; Yasuda et al., 2019). Several drugs targeting molecules associated with Th17 cell differentiation and maintenance are known to have clinical efficacy in treating Th17-associated diseases (Spandana et al., 2013; Akash and Jadhav, 2022).

The effect of TC on individual purified and polarized T cell subsets has not been studied to date. This study aims to fill the literature gap by understanding the direct immunomodulatory activity of TC on primary CD4^+^ T cells, i.e., Th1, Th17, iTreg cells, and elucidating the mechanism of action of TC on *in vitro* polarized Th17 cells through in-depth genome-wide expression profiling, and studying the binding efficiency of various components of TC with proteins involved in Th17 differentiation and proliferation through *in silico* analysis.

## Materials and methods

### *T.cordifolia* extract preparation

*Tinospora cordifolia* whole water-soluble extract, prepared as per the Ayurvedic Pharmacopoeia of India standards by GMP certified manufacturer, was commercially procured. The crude aqueous extract of *T.cordifolia* (AETC) was reconstituted by mixing 100 mg of the lyophilized plant material with 1 ml Milli-Q, mixed well, and kept at room temperature for 1 h followed by overnight incubation at 4°C. The next day, the aqueous extract was filtered through a 0.45 µm filter and used for assays at different concentrations.

### Chemical profiling of *T.cordifolia* by LC-MS

The chemical fingerprinting of the commercially procured TC was performed using the LC-MS technique at SAIF, CSIR-CDRI, Lucknow, India (Supplementary Data Sheet S1 in Supplementary Material). Briefly, the analysis was carried out using an Agilent 1200 HPLC system interfaced with an Agilent 6520 hybrid quadrupole time of flight mass spectrometer (Agilent Technologies, Santa Clara, CA, United States). 1200 HPLC system was equipped with a quaternary pump (G1311A), online vacuum degasser (G1322A), autosampler (G1329A), and a diode-array detector (G1315D). The separation of the compounds was carried out on a Thermo Scientific Accucore C18 column (150 mm × 2.1 mm, 2.6 μm) operated at 25°C. The mobile phase consisted of 5 mM ammonium acetate (D) and acetonitrile (B) and was delivered at a flow rate of 0.25 ml/min under a gradient program. The gradient system of *T.cordifolia* extract consisted of 0.1% formic acid in water (A) and acetonitrile (B), and the gradient system was 0.1–1 min, 100%–100% B; 1–12 min, 100%–30% B; 12–14 min, 30%–30% B; 14–16 min, 30%–100% B; 16–20 min, 100%–100% B. The sample injection volume was 5 μl. The diode array detector was set to monitor at 254 and 280 nm, and the online UV spectra were recorded in the scanning range of 190–400 nm. The mass spectrometer was operated in positive electrospray ionization mode, and mass spectra were recorded by scanning the mass range of m/z 50–1500 in MS positive mode. Nitrogen was used as drying, nebulizing, and collision gas. The drying gas flow rate was 12 L/min. The heated capillary temperature was set to 350°C, and the nebulizer pressure was at 45 psi. The source parameters capillary voltage (VCap), fragmentor, skimmer, and octapole voltages were set to 3500 V, 175 V, 65 V, and 750 V, respectively. The accurate mass data of the molecular ions were processed through the Mass Hunter version B 04.00 (Agilent Technology Santa Clara, CA, United States).

### Mice

C57BL/6 mice were obtained from the animal facility of CSIR- Institute of Genomics and Integrative Biology (IGIB), New Delhi, India. Mice aged between 7 and 10 weeks were used for all experiments. All mice were bred and maintained under specific pathogen-free conditions at the animal facility of IGIB, as per the Committee for the Purpose of Control and Supervision of Experiments on Animals (CPCSEA). All animal experiments were performed in compliance with protocols approved by the Institutional Animal Ethics Committee of CSIR-IGIB, Delhi, India.

### Reagents

Monoclonal antibodies specific to the following mouse antigens were purchased from Thermo Fisher Scientific, eBioscience, United States: CD4 Alexa488 (GK1.5), Foxp3 eFluor450 (FJK-16s), IL-17A APC (eBio17B7), CD8 PE (Ly-2), CD25 PE (PC61), and IFN gamma PE (XMG1.2).

### Cell isolation

#### CD8^−^CD25^−^ cell isolation

CD8^−^CD25^−^ cells were isolated using the MACS column technique. Briefly, single-cell suspension isolated from the spleen and lymph nodes of mice was resuspended in the MACS buffer, anti-CD8 PE and anti-CD25 PE antibodies were added and incubated for 15 min at 4°C. Cells were washed and resuspended in MACS buffer, and anti-PE microbeads, mixed and incubated for 20 min at 4°C. Cells were subjected to MACS MS columns in a MACS separator, and the flow-through consisting of CD8^−^CD25^−^ cells were collected.

#### CD4^+^CD25^−^ T cell isolation

CD4^+^ T cells were isolated from the spleens and lymph nodes of mice by enrichment with the Dynal Mouse CD4 Negative Isolation Kit (Life Technologies, United States). The isolated CD4^+^ T cells were incubated with an anti-CD25 PE antibody to isolate CD4^+^CD25^−^ T cells. CD4^+^CD25^−^ T cells were isolated using the MACS column, as mentioned above. The purity of CD4^+^CD25^−^ T cells was >90%.

### Th1, Th17, and iTreg cell culture

TexMACS™ RPMI 1640 GlutaMAX (Miltenyi Biotech, Germany) medium was used for CD4, iTreg, and Th1 cell cultures, and IMDM (Gibco, United States) for Th17 cell cultures. The medium was supplemented with 10% heat-inactivated FCS (Gibco, United States), 500U penicillin-streptomycin (Life Technologies, United States), and 50 µM β-mercaptoethanol (Life Technologies, United States). For Th17 cell induction, 1–1.5 × 10^5^ CD4^+^CD25^−^ T cells were cultured for 4 days with plate-bound anti-CD3ε (10 µg/ml, clone 145-2C11; Bio X Cell), anti-CD28 (1 µg/ml, clone 37.51; Bio X Cell), anti-IFN-γ (5 µg/ml, clone XMG1.2; Bio X Cell), anti-IL-4 (5 µg/ml, clone 11B11; Bio X Cell), rhTGFβ1 (2 ng/ml; Peprotech), rmIL-6 (5 ng/ml; Peprotech) and rmIL-1β (50 ng/ml; Peprotech). For iTreg cell induction, 5 × 10^4^ CD4^+^CD25^−^ T cells were cultured for 4 days in the presence of plate-bound anti-CD3ε (5 µg/ml), anti-CD28 (1 µg/ml), rhIL-2 (200 U/mL; Roche Applied Science, Germany) and rhTGFβ1 (2 ng/ml). On day 2, rhIL-2 (200 U/mL) was added again. For Th1 cell culture differentiation, 1–1.5 × 10^5^ CD4^+^CD25^−^ T cells were cultured for 5 days with plate-bound anti-CD3ε (10 µg/ml, clone 145-2C11; Bio X Cell), anti-CD28 (1 µg/ml, clone 37.51; Bio X Cell), rmIL-12 (5 µg/ml; Peprotech), anti-IL-4 (5 µg/ml, clone 11B11; Bio X Cell). The cultured cells were treated with 500, 1000, 1500, and 2000 µg/ml of AETC or Milli-Q as a vehicle control on day 0.

### T cell proliferation assay

CD4^+^CD25^−^ T cells were isolated as mentioned above and labeled using 5 µM CellTrace Violet Cell Proliferation Kit (Life Technologies, United States). Briefly, the isolated CD4^+^CD25^−^ T cells were washed and resuspended in warm PBS. Naive T cells were labeled using 5 µM CellTrace Violet and incubated in the dark for 15 min in a 37°C water bath. The labeled cells were centrifuged, washed with a warm culture medium, and resuspended as 1 × 10^6^ cells/mL. The cells were either stimulated with anti-CD3ε and anti-CD28 and cultured as such or differentiated to Th17 cells following the Th17 differentiation protocol mentioned above. The cultured cells were treated with 500, 1000, 1500, and 2000 µg/ml of AETC or Milli-Q as a vehicle control on day 0. On day 4, cells were harvested and stained with ethidium monoazide (EMA), followed by surface staining with anti-CD4. Cell proliferation was determined by quantifying the dilution of CellTrace Violet on CD4^+^ live cells by flow cytometry using LSRII (Becton Dickinson, United States) or FACSAria (Becton Dickinson, United States).

### Flow cytometry

For analysis of surface markers, cells were stained with the specific surface marker for each phenotype in PBS containing 0.25% BSA and 0.02% azide. Dead cells were excluded by EMA (Life Technologies, United States). For intracellular cytokine staining, cells were stimulated with phorbol 12-myristate 13-acetate (0.1 µg/ml; Sigma-Aldrich) and ionomycin (1 µg/ml; Sigma-Aldrich) for 2 h, followed by Brefeldin A (5 µg/ml; eBioscience) for 2 h and stained using the Foxp3/Transcription Factor Fixation/Permeabilization Kit (eBioscience, United States) according to the manufacturer’s instructions. Cells were acquired on FACSAria (Becton Dickinson, United States) or LSR II (Becton Dickinson, United States), and data were analyzed with FlowJo software (Tree Star, Version 9, United States).

### Cytotoxicity assay

CD4^+^CD25^−^ T cell were isolated from splenocytes as mentioned above and were cultured with 500, 1000, 1500, and 2000 µg/ml of AETC or Milli-Q as a vehicle control on day 0 for 24 and 96 h. Cells were washed with PBS containing 0.25% BSA and 0.02% azide and stained with a live dead stain, either propidium iodide (PI) or EMA (Sigma, United States), to analyze cell viability using the flow cytometry technique. Cells were acquired on FACSAria (Becton Dickinson, United States) or LSR II (Becton Dickinson, United States), and data were analyzed with FlowJo software (Tree Star, Version 9, United States).

### Cell cycle

Th17 cell culture was performed as mentioned above, and cultured cells were treated with 500, 1000, 1500, and 2000 µg/ml of AETC or Milli-Q as a vehicle control on day 0. On day 4, cells were washed with PBS and fixed in 70% ethanol at 4°C overnight. Further, the cells were centrifuged at 380 × g for 5 min at room temperature. The supernatant (ethanol) was removed, and the cells were resuspended in PBS and centrifuged again. The supernatant was removed, the cell pellet was resuspended in PBS, and 10 µl of RNase A (10 mg/ml; Thermo Fisher Scientific, United States) was added. The samples were incubated for 4 h at 37°C. Finally, 10 µl of PI (1 mg/ml) was added, and the cells were incubated for an additional 2–3 min in the dark at 37°C. The cell cycle distribution was determined by flow cytometry for DNA content using LSR II (Becton Dickinson, United States). The data were finally presented as percentages of the cells in the G0/G1, S, and G2/M phases using Flowjo software.

### RNA isolation and whole transcriptome analysis

Total RNA from Th17 cultured cells, treated with 500, 1000, and 1500 µg/ml of AETC or Milli-Q as a vehicle control on day 0, was isolated as per the manufacturer’s protocol for the RNAeasy Mini Kit (Qiagen, Valencia, CA). Isolated RNA integrity was checked on a 1% agarose gel followed by Nanodrop quantification (ND1000, Nanodrop Technologies, United States). The Affymetrix Gene Chip MTA 1.0 array was used for genome-wide expression analysis according to the manufacturer’s instructions. In brief, total RNA, 250 ng for each sample, was quantified and hybridized to microarray chips following the sequential steps described in the protocol. After hybridization, microarray chips were washed and stained using the Affymetrix Gene Chip Fluidics Station 450. The stained chips were finally scanned using an Affymetrix Gene Chip Scanner 3000 (Affymetrix, CA, United States). The signal values were further evaluated using the Affymetrix^®^ GeneChip™ Command Console software. Raw data was automatically extracted using the Affymetrix data extraction protocol in the AffymetrixGeneChip^®^ Command Console^®^ Software (AGCC). A comparative study between the vehicle control and the AETC treated samples was done using the Transcriptome Analysis Console (TAC) software. TAC was used to import. CEL files, perform normalization, remove batch effects, and analyze differentially expressed genes. The genes with absolute log2 fold change ≥2 or ≤2 and *p*-value < .05 were considered differentially expressed. The raw data files have been submitted to Gene Expression Omnibus (GEO) with the accession number GSE201104.

### Functional enrichment and gene set enrichment analysis

Functional analysis was performed using the Enrichr (maayanlab.cloud/Enrichr) tool. For gene ontology and pathway analysis, we examined gene enrichment in Biological Processes, KEGG, BioPlanet, and WikiPathway. The gene set enrichment was considered significant when the *p*-value was < .05 in the Enrichr tool. To identify specific gene sets modulated by AETC, Gene set enrichment analysis (GSEA) was performed using the GSEA (gsea-msigdb.org) tool. A pre-ranked analysis was done using a list of genes ordered from most positive to most negative gene expression. The hallmark genes dataset was selected for the query, and datasets with >500 and <15 genes were excluded. A minimum cut-off of *p*-value < .05, and corrected for FDR <25% was set for the output.

### Reverse transcription and quantitative real-time PCR

Total RNA from Th17 cultured cells, treated with 1500 µg/ml of AETC or Milli-Q as a vehicle control on day 0, was isolated according to the manufacturer’s protocol for the RNAeasy Mini Kit (Qiagen, Valencia, CA). Complementary DNA was synthesized using a High-Capacity cDNA Reverse Transcription Kit (Thermo Scientific, United States) according to the manufacturer’s instructions. Quantitative PCRs (qPCRs) were performed using SYBR Green (Thermo Fisher Scientific, United States). Primers used to quantify RORC, HIF1-α, IL-23R, CD3, IL-17A, IL-17R, IL-7R, SLC2A3, GLUT1, IFN-γ, PHD2, HK2 and VEGF were as shown in Table 1.

**TABLE 1:** The primer sequences used in this study.

Gene	Forward primer sequence (5′-3′)	Reverse primer sequence (5′-3′)
RORC	5′-AGTCGTCCTAGTCAGAATG-3′	5′-ATGTTCCACTCTCCTCTTC-3′
HIF1-α	5′-TCAGCAACGTGGAAGGTGCT-3′	5′- AATCAGCACCAAGCACGTCAT-3′
IL-23R	5′-GCTCGGATTTGGTATAAAGG-3′	5′-ACTTGGTATCTATGTAGGTAGG-3′
CD3	5′-GCTCCAGGATTTCTCGGAAGTC-3′	5′- ATGGCTACTGCTGTCAGGTCCA-3′
IL-17A	5′-AGGCAGCAGCGATCATCC-3′	5′-GTGGAACGGTTGAGGTAGTC-3′
IL-17R	5′-CTGTATGACCTGGAGGCTTTCTG-3′	5′-CGAGTAGACGATCCAGACCTTC-3′
IL-7R	5′-CACAGCCAGTTGGAAGTGGATG-3′	5′-GGCATTTCACTCGTAAAAGAGCC-3′
SLC2A3	5′-CCGCTTCTCATCTCCATTGTCC-3′	5′- CCTGCTCCAATCGTGGCATAGA-3′
GLUT1	5′-GCTTCTCCAACTGGACCTCAAAC-3′	5′- ACGAGGAGCACCGTGAAGATGA-3′
IFN-γ	5′-CTGAGACAATGAACGCTACAC-3′	5′-TCCACATCTATGCCACTTGAG-3′
PHD2	5′-GACCGGCGTAACCCTCATG-3′	5′- TTGCTGACTGAATTGGGCTTG-3′
HK2	5′-ACTCCAGACGGTACAGAGAAA-3′	5′-TCTTGTTATGCATCTCTACGC-3′
VEGF	5′-CTGCTGTAACGATGAAGCCCTG-3′	5′-GCTGTAGGAAGCTCATCTCTCC-3′
ACTB	5′-GTGAAAAGATGACCCAGATCAT-3′	5′-TGTGGTACGACCAGAGGCATA-3′

Primer sequences for CD3, IL-17R, IL-7R, SLC2A3, VEGF, and PHD2 were obtained through OriGene. The levels of *RORC, HIF1-α, IL-23R, CD3, IL-17A, IL-17R, IL-7R, SLC2A3, GLUT1, IFN-γ, PHD2, HK,* and *VEGF* were determined by evaluating the threshold cycle (Ct) of the target gene after normalization against the Ct value of *ACTB.*

### Molecular docking

The compounds of TC were identified from a literature search, and 69 compounds were found (Supplementary Table S5 in Supplementary Material) (Spandana et al., 2013; Akash and Jadhav, 2022). The 3D structures of all 69 TC compounds were obtained from PubChem (Kim et al., 2021). All the 69 compounds were treated as ligand molecules and docked against 15 of the T cell-mediated immune response proteins, namely TNF-α (2AZF), TNF-β (2TGI), IFN-γ (1FG9), IL-6 (1ALU), IL-17 (4HR9), IL-21 (2OQP), IL-23 (5MZV), IL-6R (1N26), IL-7R (3DI2), IL-17R (3JVF), IL-23R (5MZV), TGFβR (1PY5), IL-1β (4G6J), IL-2 (1Z92) and IL-12 (1Z92). The 3D crystal structures of all the proteins were extracted from the Protein Data Bank (https://www.rcsb.org). The active regions of all proteins were identified using the COACH meta-server, and these results were compared and validated with the results obtained from the CASTp web server (Yang et al., 2013; Tian et al., 2018). The impact on the T cell target proteins was investigated for the compounds of TC by molecular docking studies using the Schrodinger suite (Maestro) (Friesner et al., 2006). The T cell target proteins were prepared using the protein preparation wizard in the Schrodinger suite (Maestro), which included optimization followed by minimization of heavy atoms of proteins. The energy-minimized 3D structures of all the ligands of TC active compounds were prepared using LigPrep. The best pose of ligands that fit well in the protein cavity was carried out using the OPLS4 force field with the Glide package in Extra Precision (XP) mode. Further molecular docking procedures were carried out using the Glide package of the Schrodinger suite (Maestro). The interactions of the docked complex in the 2D interaction diagram were accessed using Discovery Studio Visualizer 3 (Accelrys Software Inc., United States 2010).

### Statistical analysis

Student’s t-test was used to assess the differences between the AETC treated and vehicle control groups. Analysis and graphs were created using GraphPad Prism Software (version 8). Data are presented as mean ± SEM or mean ± SD with *p* values considered significant as *p < .05, **p < .01, and ***p < .001.

## Results

### Characterization of compounds in *T.cordifolia* by LC-MS analysis

Compounds reticuline (Rt 10.62) m/z 330 [M + H]+, tinocordiside (Rt 8.14) m/z 419 [M + Na]+, 20β-Hydroxyecdysone (Rt 10.83) m/z 481 [M + H]+, cordifolioside A (Rt 5.82) m/z 503 [M + Na]+, tinosporinone (Rt 5.24) m/z 342 [M]+, 1,2-dihydroxyaporphine (R)-form, O1-Me, N-de-Me, N-formyl, 2-O-[β-d-glucopyranosyl-(1→2)-β-d-glucopyranoside] (Rt 7.86) m/z 601 [M + H2O]+, jatrorrhizine (Rt 10.2.) m/z 338 [M]+, cordifolide A (Rt 8.48) m/z 621 [M + Na]+ (Supplementary Data Sheet S1 in Supplementary Material) were identified based on LC-MS results. This analysis provides information on the presence of major secondary metabolites such as reticuline, tinocordiside, 20β-Hydroxyecdysone, cordifolioside A, tinosporinone, jatrorrhizine, and cordifolide in the complex mixture of TC used for the study.

### *T.cordifolia* aqueous extract does not exhibit cytotoxicity in primary CD4^+^ T cells

**FIGURE 1:**
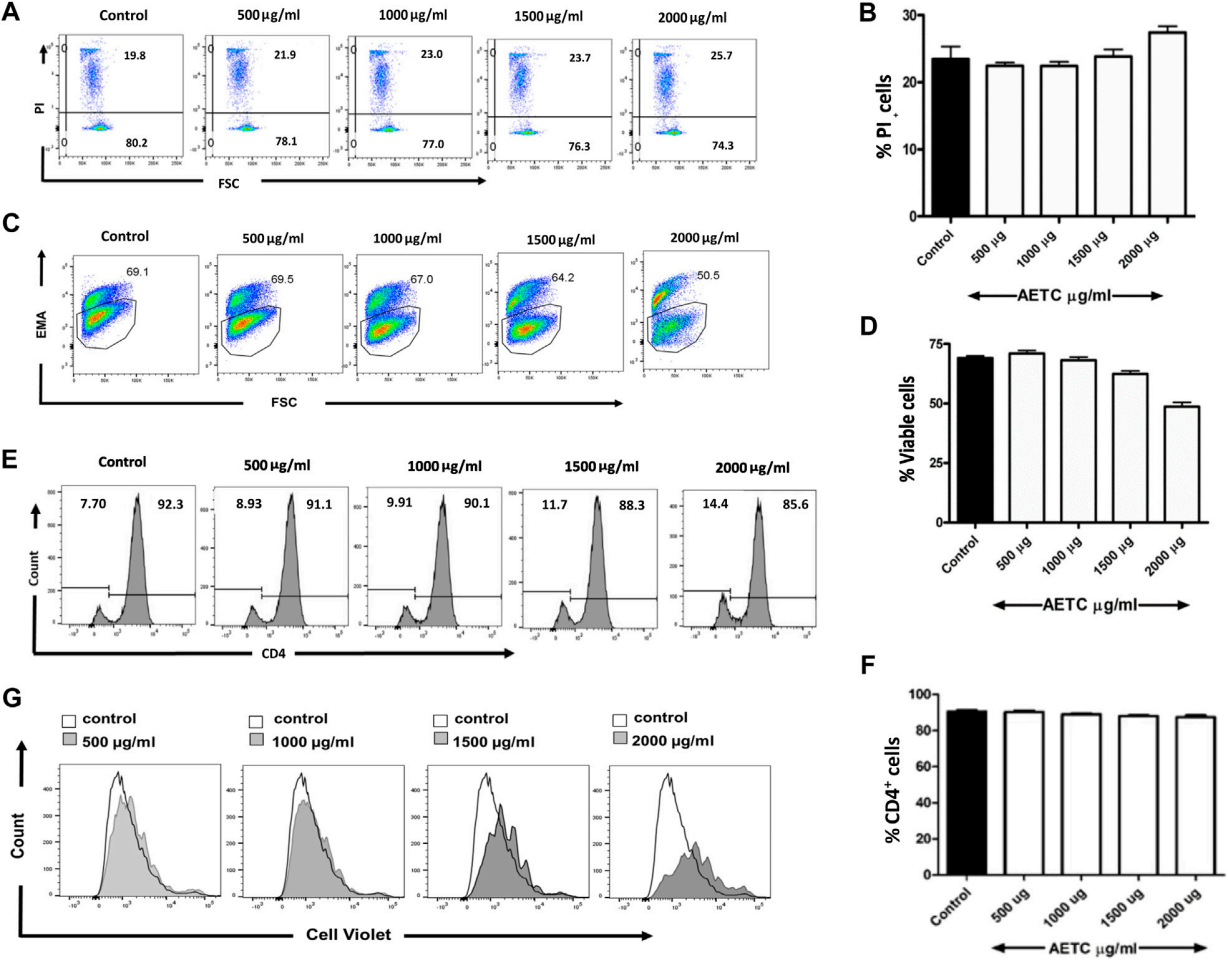
*T. cordifolia* aqueous extract does not exhibit cytotoxicity in primary CD4^+^ T cells. Naive T cells were isolated from C57BL/6 mice, stimulated with anti-CD3 and anti-CD28 mAb, and cultured in the presence of AETC (500–2000 µg/ml) or vehicle control for 24 and 96 h. Representative flow cytometry showing the percentage of **(A)** PI positive cells following the culture of naive T cells for 24 h and **(C)** EMA positive cells for 96 h in the presence of AETC (500–2000 µg/ml) or vehicle control. Bar graphs represent the quantification of flow cytometry data for **(B)** 24 h and **(D)** 96 h of AETC treatment or vehicle control. **(E)** Histogram and **(F)** bar diagram representing cell surface expression of CD4 molecule on naive T cells, stimulated with anti-CD3 and anti-CD28 mAb and cultured in the presence of AETC (500–2000 µg/ml) or vehicle control for 96 h **(G)** The proliferation of AETC (500–2000 µg/ml) or vehicle control as assessed by the cell proliferation dye CellTrace violet. Cells were stained for CD4 and gated on live CD4^+^ T cells. Results are representative of three independent experiments. Error bars represent the mean ± SEM of triplicates. *p < .05, **p < .01 and ***p < .001. Student’s *t*-test (vehicle control *versus* AETC).

The naive CD4^+^ T cells were treated with increasing doses of AETC (500, 1000, 1500, and 2000 µg/ml) in the presence of stimulation (anti-CD3 and anti-CD28 mAb) to assess the cytotoxic effect of AETC on non-polarized CD4^+^ T cells. We found no cytotoxic activity of AETC in CD4^+^ T cell cultures at doses ranging between 500 and 2000 μg/ml, compared with the vehicle control at 24 h of incubation (Figures 1A, B). However, moderate cytotoxicity was observed on incubation of CD4^+^ T cells for 96 h with AETC at a higher concentration, i.e., 2000 μg/ml, when compared to the vehicle control (Figures 1C, D). Hence, we proceeded with studying the activity of AETC on the development of T cells *in vitro* at doses ranging from 500 to 2000 μg/ml.

Next, we looked at the effect of AETC on the expression pattern of CD4 molecules on naive CD4^+^ T cells. Naive CD4^+^ T cells stimulated with anti-CD3 and anti-CD28 mAb under non-polarizing conditions and treated with AETC did not exhibit significant inhibition of the CD4 surface molecule on CD4^+^ T cells (Figures 1E, F). Interestingly, inhibition of proliferation of naive CD4^+^ T cells in the anti-CD3 and anti-CD28 mAb stimulated T cell culture was observed above 1500 µg/ml compared to the vehicle control (Figure 1G).

### *T.cordifolia* extract reduces Th17 cell frequency and exhibits anti-proliferative activity

**FIGURE 2:**
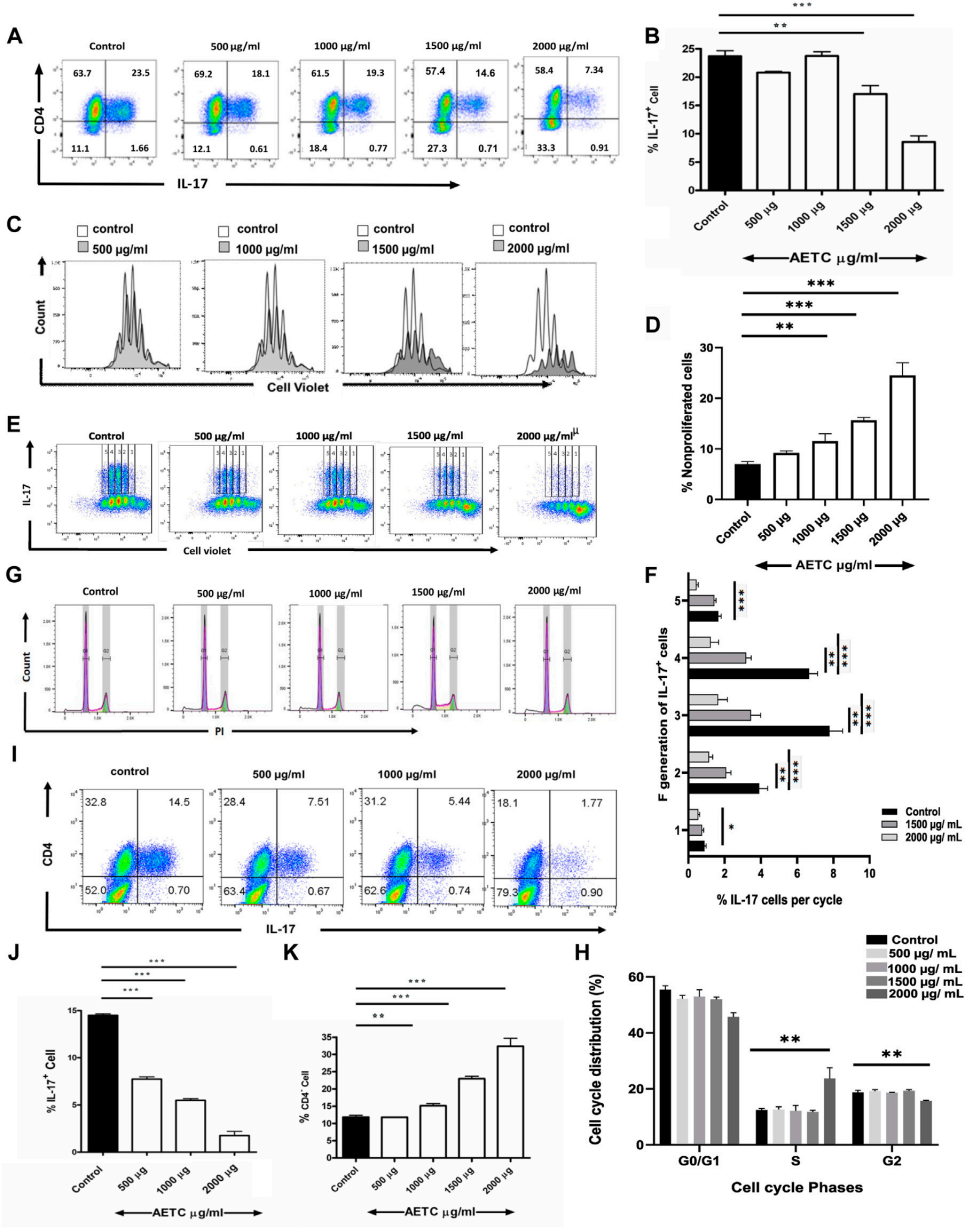
*T. cordifolia* extract reduces Th17 cell frequency, exhibits anti-proliferative activity on CD4^+^ T cells, and promotes the proliferation of CD4^−^ cells. **(A,B)** Frequency of IL-17^+^ cells following culture of naive T cells for 4 days under Th17-polarizing conditions in the presence of AETC (500–2000 µg/ml) or vehicle control. Cells were stained for intracellular IL-17 and were gated on live cells. **(A)** Representative flow cytometry. Numbers represent the percentage of CD4^+^ and IL-17^+^ T cells among total live cells. **(B)** Percentage of IL-17^+^ cells among total live CD4^+^ T cells cultured with AETC (500–2000 µg/ml) or vehicle control. **(C,D)** The proliferation of AETC (500–2000 µg/ml) or vehicle control as assessed by the cell proliferation dye CellTrace violet. Cells were stained for intracellular IL-17 and gated on live CD4^+^ T cells. **(C)** The histogram represents an overlay of cell count for vehicle control *versus* AETC treated groups. **(D)** Bar graphs represent the percentage of nonproliferated cells. **(E,F)**, **(E)** Representative flow cytometry data with 1-5 proliferation cycles and **(F)** bar graphs represent the percentage of IL-17^+^ cells among total live CD4^+^ T cells in each proliferation cycle. **(G,H) (G)** Histogram representing cell cycle analysis of Th17 polarized cells treated with 500–2000 µg/ml of AETC and vehicle control. **(H)** Bar graph showing the percentage of polarized Th17 cells in G0/G1, S, and G2 phases in naive T cells cultured for 4 days under Th17-polarizing conditions in the presence of AETC (500–2000 µg/ml) or vehicle control. **(I,J)** Frequency of IL-17^+^ cells following the culture of CD8^−^CD25^−^ splenocytes for 4 days under Th17-polarizing conditions in the presence of AETC (500–2000 µg/ml) or vehicle control. Cells were stained for intracellular IL-17 and were gated on live cells. **(I)** Representative flow cytometry. Numbers represent the percentage of CD4^+^ and IL-17^+^ positive T cells among total live cells. **(J)** Percentage of IL-17^+^ cells among total live cells cultured with AETC (500–2000 µg/ml) or vehicle control. **(K)** The bar graph represents the percentage of CD4^−^ cells among total live cells following the culture of naive T cells for 4 days under Th17-polarizing conditions in the presence of AETC (500–2000 µg/ml) or vehicle control. Results are representative of three independent **(A–K)** experiments. Error bars represent the mean ± SEM of triplicates. *p < .05, **p < .01 and ***p < .001. Student’s *t*-test (vehicle control *versus* AETC).

*In vitro* Th17 cell differentiation assay was carried out to study the activity of AETC on the generation of Th17 cells. Naive mouse CD4^+^ T cells, when cultured *in vitro* under Th17-polarizing conditions in the presence of AETC, exhibited a reduction in the frequency of IL-17 producing Th17 cells in a dose-dependent manner at concentrations above 1500 μg/ml (Figures 2A, B). Analysis of the live CD4^+^ T cell population under the same polarizing condition revealed that AETC negatively regulates the proliferation of these subsets at doses above 1000 µg/ml compared to the vehicle control (Figures 2C, D). Moreover, each proliferation cycle analysis showed that the frequency of IL-17^+^ cells was lower in AETC treated cells than in vehicle control at different generations (Figures 2E, F). Additionally, to understand the cause of the reduction of CD4^+^ cells, cell cycle analysis was performed, which showed some extent of cell cycle arrest in the G2 phase only at the highest dose of 2000 μg/ml (Figures 2G, H).

To know whether AETC exhibits its regulatory activity in a complex cell system, we performed a cell culture with CD8^−^CD25^−^ splenocytes in a Th17-polarizing condition in the presence of AETC. We found that AETC significantly reduced the production of pro-inflammatory cytokine IL-17 in a dose-dependent manner above 500 μg/ml (Figures 2I, J).

In the Th17-polarizing conditions, in addition to reduced proliferation, a significant decrease in the frequency of CD4^+^ T cells was observed above 1000 µg/ml of AETC, accompanied by an increase in the frequency of CD4^−^ cells (Figures 2A, K).

### *T.cordifolia* extract downregulates transcriptional expression of molecules associated with Th17 cell development

**FIGURE 3:**
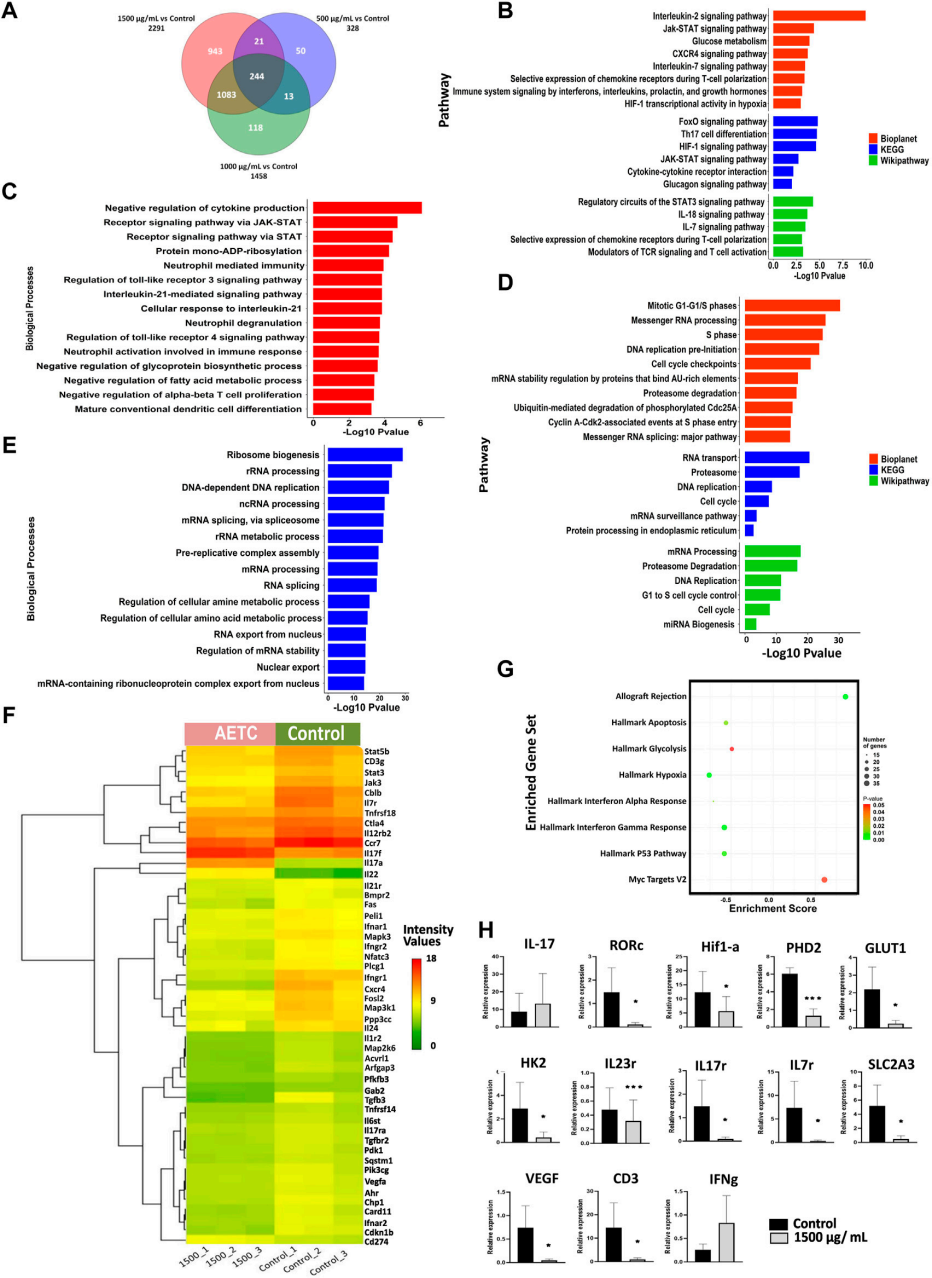
Differential gene expression pattern contributing towards inhibition of Th17 cell differentiation on AETC treatment. **(A)** Venn diagram representing the total number of differentially expressed genes and common genes at different doses of AETC. **(B)** Enriched pathways in downregulated and **(D)** upregulated genes after AETC treatment at 1500 μg/ml compared to vehicle control. **(C)** Enriched biological processes in downregulated and **(E)** upregulated genes after AETC treatment at 1500 μg/ml compared to vehicle control. **(F)** Heatmap representing genes differentially regulated and involved in Th17 cell differentiation, HIF1 signaling, Cytokine-cytokine receptor interaction, T cell receptor signaling pathway, Signaling by interleukins, and Interleukin-7 signaling pathway. The differentially regulated genes with FC ≥ 2 or ≤2 and a *p*-value < .05 were selected for graphs. **(G)** Gene set enrichment analysis for genes differentially expressed in response to AETC at 1500 μg/ml in Th17 cell culture with cut-off values of *p*-value < .05 and FDR < 25%. **(H)** Expression of Th17-related genes in AETC treated naive T cells cultured under Th17-polarizing conditions was assessed by quantitative PCR. The data shown are combined results from three experiments. Error bars show mean ± SD. *, *p* < .05, **, *p* < .01, ***, *p* < .001.

To understand the molecular mechanism of TC inhibition, transcriptomic analysis of naive CD4^+^ T cells, differentiated *in vitro* under Th17-polarizing conditions in the presence of AETC at different doses (1500, 1000, and 500 µg/ml), was performed. AETC treatment at 1500, 1000, and 500 µg/ml showed an upregulation of 1406, 771, and 229 genes, respectively (Supplementary Table S1 in Supplementary Material). While 885, 687, and 99 genes were downregulated at 1500, 1000, and 500 µg/ml of AETC treatment, respectively, (Supplementary Table S1 in Supplementary Material) compared to the vehicle control. A total of 244 genes were common at all three concentrations, i.e., 500, 1000, and 1500 µg/ml of AETC when compared (Figure 3A). We observed enrichment of pathways like Th17 differentiation, JAK-STAT signaling pathway, HIF1 signaling pathway, IL-7 signaling pathway, IL-2 signaling pathway, glucose metabolism, FoxO signaling pathway, cytokine-cytokine receptor interaction in the 1500 µg/ml of AETC downregulated genes (Figure 3B). In accordance with the signaling cascade, biological processes such as negative regulation of cytokine production, receptor signaling pathway *via* JAK-STAT, neutrophil-mediated immunity and neutrophil degranulation, interleukin-mediated signaling pathway, cellular response to interleukin-21, regulation of toll-like receptor 3 and 4 signaling pathway were enriched in 1500 µg/ml of AETC downregulated genes (Figure 3C; Supplementary Table S2 in Supplementary Material). At 1000 µg/ml of AETC, downregulated genes contained gene sets enriched in pathways involved in Th17 cell differentiation, FoxO signaling pathway, selective expression of chemokine receptors during T-cell polarization, Interleukin-2 signaling pathway, HIF1 transcriptional activity in hypoxia (Supplementary Table S3 in Supplementary Material). Th17 cell differentiation, FoxO signaling pathway, Interleukin-2 signaling pathway, and IL-7 signaling pathways were significantly enriched at 1500, and 1000 µg/ml of AETC treated Th17 cells. The genes *STAT5B, STAT3, AHR, TGFBR2, RARA*, *IL-21R, IL-6ST, JAK3, MAPK3, VEGFA,* and *SLC2A3*, important for Th17 cell differentiation and homeostasis, were downregulated (Figure 3F). Interestingly, the expression of *IL-17A, IL-17F, IL-1A, IL-2, IL-22, IL-3,* and *IL-9,* important cytokines secreted by activated T cells and Th17 cells, were observed to be upregulated (Figure 3F; Supplementary Table S1 in Supplementary Material). Genes involved in interleukin signaling pathway mainly *IL-2*, *IL-7, ITGB2, IL-24, CCR7, PARP8, TGFBR2, HDAC4, CXCR4, STAT4, TNFRSF14, IL-12RB2, FOSL2, BCL6, CD27 FAS, CD68, IL-7R, PIK3CG, CDKN1B, IL-1R2,* were significantly underexpressed in the AETC treated Th17 cells (Figure 3F; Supplementary Table S1 in Supplementary Material). *MAP3K1, NFATC3, CD3G, ARFGAP3, PIK3CG, CTLA4, PLCG1, CARD11, PDK1*, genes of the T cell receptor signaling pathway, were also downregulated (Figure 3F; Supplementary Table S1 in Supplementary Material). The upregulated cascade of genes included pathways and processes such as ribosome biogenesis, RNA processing, splicing, and cell cycle (Figures 3D, E; Supplementary Table S4 in Supplementary Material). Gene set enrichment analysis of differentially expressed genes at 1500 µg/ml of AETC compared with vehicle control using the Hallmark database exhibited two positively and six negatively enriched gene sets (FDR <25%) (Figure 3G; Supplementary Tables S2, S4 in Supplementary Material). Significant negative enrichment of gene sets of processes involved in the differentiation of Th17 cells was observed, which was in line with the functional analysis. We further confirmed the microarray results with RT-PCR. AETC treated Th17 polarized cells showed reduced expression of *IL-17R, IL-7R, SLC2A3,* and *CD3* and enhanced expression of *IL-17* and *IFN-γ*. Additionally, we studied genes *RORC, HIF1-α, PHD2, GLUT1, HK2, IL-23R,* and *VEGF* relevant for Th17 polarization and development. Genes *RORC, HIF1-α, PHD2, GLUT1, HK2, IL-23R,* and *VEGF* exhibited reduced expression at 1500 μg/ml of AETC treatment relative to the vehicle control (Figure 3H).

### *T.cordifolia* extract downregulates IFN-γ production in Th1 cells

**FIGURE 4:**
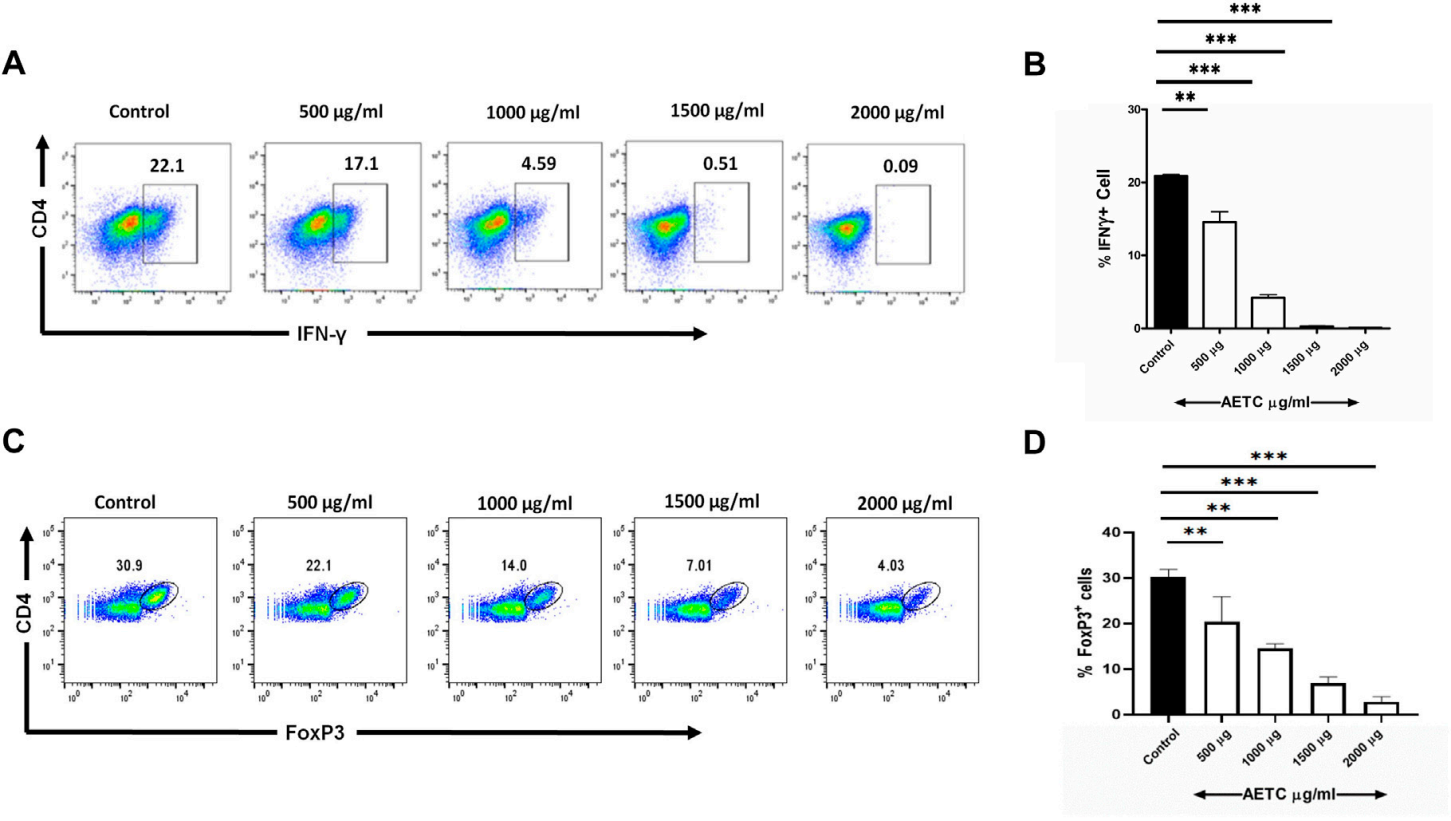
*T. cordifolia* extract downregulates IFN-γ production in Th1 cells and FoxP3 expression in iTreg cells. **(A,B)** Frequency of IFN-**γ** ^+^ cells following the culture of naive T cells for 5 days under Th1-polarizing conditions in the presence of AETC (500–2000 µg/ml) or vehicle control. Cells were stained for intracellular IFN-**γ** and were gated on live cells. **(A)** Representative flow cytometry. Numbers represent the percentage of IFN-**γ** ^+^ T cells among total live cells. **(B)** Percentage of IFN-**γ** ^+^ T cells among total live cells cultured with AETC (500–2000 µg/ml) or vehicle control. **(C,D)** Frequency of FoxP3^+^ cells following the culture of naive T cells for 4 days under iTreg polarizing conditions in the presence of AETC (500–2000 µg/ml) or vehicle control. Cells were stained for intracellular FoxP3 and were gated on live cells. **(C)** Representative flow cytometry. Numbers represent the percentage of FoxP3^+^ T cells among total live cells. **(D)** Percentage of FoxP3^+^ cells among total live cells cultured with AETC (500–2000 µg/ml) or vehicle control. Results are representative of three independent experiments. Error bars represent the mean ± SEM of triplicates. *p < .05, **p < .01 and ***p < .001. Student’s *t*-test (vehicle control *versus* AETC).

After investigating the effect of AETC on Th17 polarized naive CD4^+^ T cells, we further studied the effect of AETC on naive CD4^+^ T cells differentiated into Th1 cells. AETC significantly reduced IFN-**γ** production in naive CD4^+^ T cells induced under Th1 polarizing conditions at concentrations of 500 μg/ml and above compared to the vehicle control (Figures 4A, B).

### *T.cordifolia* extract downregulates FoxP3 expression in iTreg

Naive CD4^+^ T cells differentiated under iTreg polarizing conditions and treated with AETC exhibited a significant downregulation of FoxP3 expression at concentrations of 500 μg/ml and above compared to the vehicle control (Figures 4C, D).

### *In silico* analysis demonstrates the immunomodulatory potential of *T.cordifolia* involving proteins essential for T cell development and proliferation

**FIGURE 5:**
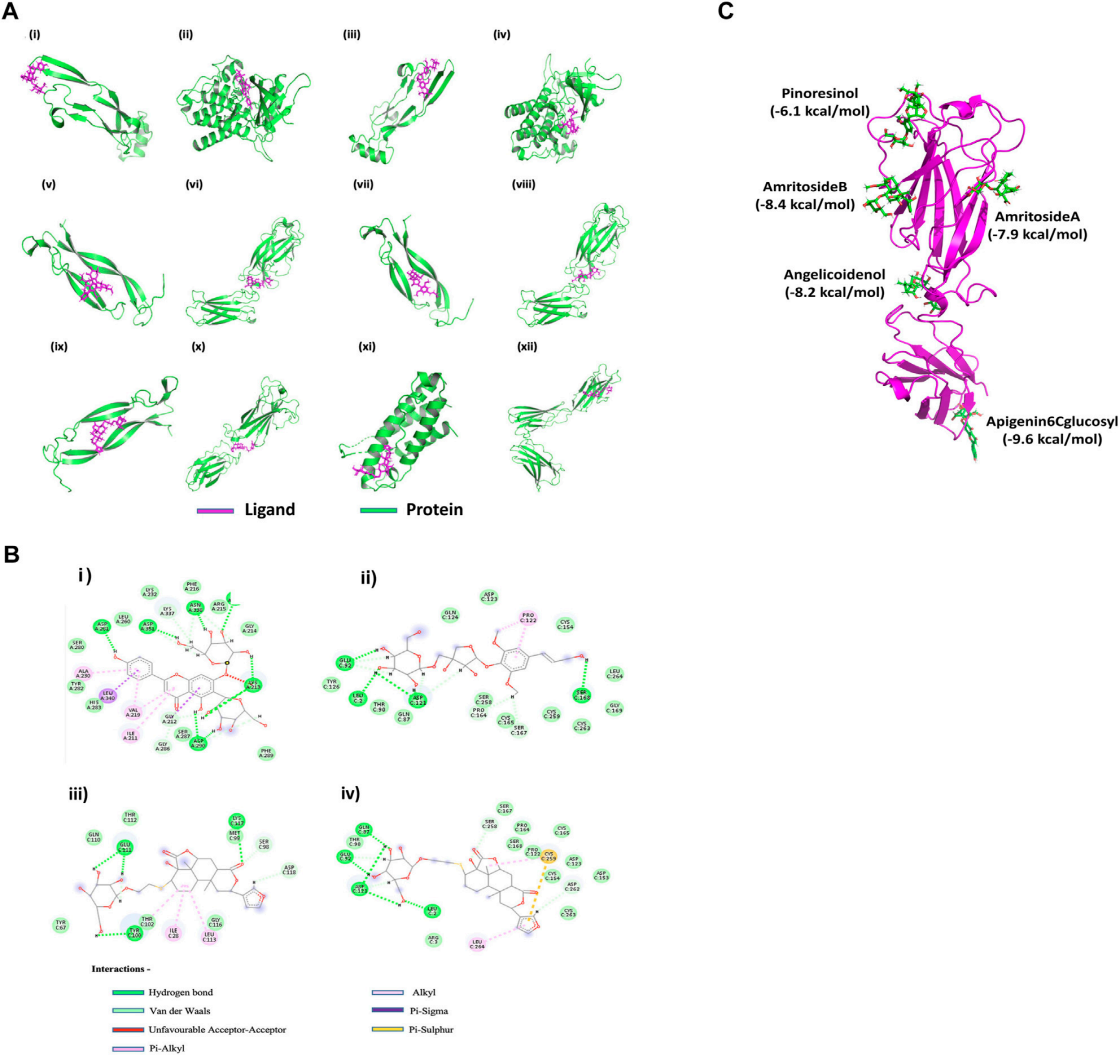
In silico effect of *T. cordifolia* secondary metabolites on Th17-associated inflammatory targets **(A)** Interaction of the best compounds Apigenin-6C-glucosyl with (i) TGFβ and (ii) TGFβR, Pinoresinol with (iii) TGFβ and (iv) TGFβR, Acetylasimilobine with (v) IL-17 and (vi) IL-17R, Cordifolioside-B with (vii) IL-17 and (viii) IL-17R, Cordifolide-A with (ix) IL-17, (x) IL-17R, (xi) IL-23 and (xii) IL-23R. **(B)** (i) Interaction plot of Apigenin-6C-glucosyl with TGFβR, (ii) Cordifolioside-B with IL-17R, (iii) Cordifolide-A with IL-17R, and (iv) Cordifolide-A with IL-23R. **(C)** Multiple docking of ligands (green) with five different cavities of IL-17R protein (Magenta). The best compounds and their corresponding energy values in five cavities are mentioned in the figure.

The secondary metabolites of TC showed a good binding affinity with the proteins and receptors involved in T cell proliferation (Supplementary Table S5 in Supplementary Material). The compound Apigenin-6-C-glucosyl has the highest binding affinity of -12.53 kcal/mol for TGFβR and -6.75 kcal/mol for TGFβ, Amritoside-A has the highest binding affinity of -8.91 kcal/mol for IL-23R, and Amritoside-B has the highest binding affinity of −6.79 kcal/mol for IL-17R. Similarly, Pinoresinol has −7.69 for IL-6, −6.95 for IL-1β, −6.86 for IL-7R, −6.59 for IL-21, and −6.56 for IL-12, Acetylasimilobine has −7.57 for IL-6R, Angelicoidenol has −7.01 for IL-2 and −6.95 for IL-17, and Secoisolariciresinol-9 has -7.09 for IL-23 (Supplementary Table S5 in Supplementary Material). In Figure 5A, the compound Apigenin-6-C-glucosyl binds to TGFβ and TGFβR with energy values of −6.75 and −12.53 kcal/mol, respectively. Pinoresinol binds to TGFβ and TGFβR with energy values −6.73 and −7.71 kcal/mol, respectively. Acetylasimilobine binds to IL-17 and IL-17R with energy values of −6.00 and −6.60 kcal/mol, respectively. Cordifolioside-B binds to IL-17 and IL-17R with energy values of −6.65 and −6.73 kcal/mol, respectively. Cordifolide-A binds to IL-17, IL-17R, IL-23, and IL-23R with energy values of −5.76, −5.62, –5.71, and −6.05 kcal/mol, respectively.

The binding affinity score of Apigenin-6-C-glucosyl and Pinoresinol for both TGFβ and TGFβR were numerically above −6 kcal/mol (Figures 5Ai–iv), considered to be a good score. Similarly, Acetylasimilobine and Cordifolioside-B have good binding affinity scores for both IL-17 and IL-17R (Figures 5Av–viii). The compound Cordifolide-A, which is exclusive to TC, showed good binding affinity (numerically above −5.5 kcal/mol) with IL-17, IL-17R, IL-23, and IL-23R (Figures 5Aix–xii).

The analysis showed Apigenin-6-C-glucosyl interacted by several hydrogen bonds with TGFβR (Figure 5Bi; Table 2), two Pi-sigma bonds formed by Leu 340 and Gly 212 of TGFβR with benzene of Apigenin-6-C-glucosyl and four Pi-Alkyl bonds formed by Ala 230, Val 219 and Ile 211 residue of TGFβR. The compound Cordifolioside-B interacted with IL-17R *via* hydrogen bonds as shown in Table 2, and alongside hydrogen bonds, two other types of bonds, Pi-Alkyl bond between Pro 122 of IL-17R with benzene of Cordifolioside-B and Alkyl bond with the Pro 122 residue were formed (Figure 5Bii). Further, Cordifolide-A showed interaction with IL-17R with five hydrogen bonds, as shown in Table 2, and also formed a Pi- Sulfur bond with Cys 259, a Pi-Alkyl bond with Leu 264, and an Alkyl bond with Cys 259 residue of IL-17R (Figure 5Biii). Cordifolide-A also showed a good interaction profile with IL-23R *via* three hydrogen bonds, two Pi-Alkyl bonds with Ile 28, Leu 113, and one Alkyl bond with Thr 102 residue of IL-23R (Figure 5Biv).

**TABLE 2:** Representation of hydrogen bonds between TC compounds with some important T cell receptor proteins.

Proteins	Ligands	Interacting residue	Interaction (D-H….A)	Hydrogen bond length in Å
TGFβR	Apigenin-6-C-glucosyl	Asp 290	(Lig.) O-H….O (Asp 290)	1.8
		Lys 213	(Lig.) O-H….O (Lys 213)	1.9
		Asp 351	(Lig.) O-H….O (Asp 351)	2.7
		Asp 281	(Lig.) O-H….O (Asp 281)	2.7
		Asn 338	(Lig.) O-H….O (Asn 338)	2.8
		Lys 213	(Lig.) O-H….N (Lys 213)	2.9
		Lys 335	(Lig.) O-H….N (Lys 335)	3.2
		Asp 290	(Lig.) O-H….O (Asp 290)	3.4
IL-17R	Cordifolioside-B	Asp 121	(Lig.) O-H….O (Asp 121)	2.6
		Asp 121	(Lig.) O-H….O (Asp 121)	2.7
		Glu 92	(Lig.) O-H….O (Glu 92)	2.8
		Leu 2	(Lig.) O-H….N (Leu 2)	2.9
		Ser 168	(Lig.) O-H….O (Ser 168)	3.1
IL-17R	Cordifolide-A	Asp 121	(Lig.) O-H….O (Asp 121)	2.9
		Asp 121	(Lig.) O-H….O (Asp 121)	2.9
		Gln 87	(Lig.) O-H….N (Gln 87)	2.9
		Glu 92	(Lig.) O-H….O (Glu 92)	3.0
		Leu 2	(Lig.) O-H….N (Leu 2)	3.1
IL-23R	Cordifolide-A	Glu 111	(Lig.) O-H….O (Glu 111)	2.6
		Glu 111	(Lig.) O-H….O (Glu 111)	2.7
		Tyr 100	(Lig.) O-H….N (Tyr 100)	3.1

A detailed study was performed to understand further the interactions of compounds in TC extract with the receptor IL-17R. The possible cavities/binding domains in the IL-17R proteins were analyzed using CASTp and COACH meta servers, and five binding regions were identified. The amino acid residues in the binding cavities are shown in Figure 5C. Further, the molecular docking study was carried out for all 69 compounds of TC with all five different cavities of IL-17R proteins. The compounds such as Pinoresinol (−6.1 kcal/mol), Amritoside A (−7.9 kcal/mol), Amritoside B (−8.4 kcal/mol), Angelicoidenol (−8.2 kcal/mol), and Apigenin 6C glycosyl (−9.6 kcal/mol) showed very high binding affinities against IL-17R protein.

## Discussion

This study identified a novel mechanism of action of TC aqueous extract, which constrains the proliferation of naive CD4^+^ T cells and the cytokine-receptor signaling pathway, resulting in reduced effector function of polarized Th17 cells. In addition, AETC was found to be non-cytotoxic until 1500 μg/ml. In previous reports, TC extract had shown increased lymphocyte proliferation in mouse spleen *in vivo* and mouse splenocytes *in vitro* (Aher and Kumar Wahi, 2012; Bala et al., 2015). However, TC aqueous extract is shown as a polyclonal B-cell activator, and the activity is directed towards B cells only while T cells remain unaffected (Chintalwar et al., 1999). Our results indicate that AETC significantly inhibits naive CD4^+^ T cell proliferation in Th17 cell culture at concentrations above 1000 μg/ml. The reduced proliferation of the CD4^+^ T cells could be a probable cause leading to a relative increase of the CD4^−^ cells in the Th17 cell culture. The population present in the CD4^−^ cell, whether a single or a mixed cell phenotype and the rationale for the increased proliferation of these unknown phenotypes needs further investigation.

The importance of IL-17 producing Th17 cells in the pathogenesis of many inflammatory and autoimmune diseases, such as multiple sclerosis, rheumatoid arthritis, psoriasis, *etc.*, is well established (Yasuda et al., 2019). This study found a dose-dependent decrease in the IL-17 producing cells at doses above 1500 μg/ml in naive cells and 500 μg/ml in the CD8^−^CD25^−^ splenocyte Th17 cell culture. In some experiments, we also observed inhibition of IL-17 even at doses of 500 μg/ml of AETC in naive cell culture, but this was inconsistent. Since CD8^−^CD25^−^ cells mimic a complex cell culture system consisting of various immune cells; it was interesting to study the activity of AETC in such a system. Our results indicated that the inhibitory activity of AETC is also maintained in the presence of other immune cells. The immune cells in CD8^−^CD25^−^ cell Th17 culture synergizing the modulatory activity of AETC remains to be studied. Analysis of separate generations of daughter cells in the AETC treated Th17 culture indicated a significant reduction in the IL-17 producing cells from the second generation at doses above 1500 μg/ml. This may indicate the development of Th17 cells being affected at an early phase by treatment with AETC. Since cell proliferation inhibition is associated with cell cycle arrest, our study did not observe a significant change in the different phases of the cell cycle until 1500 µg/ml. An increased frequency of cells was observed in the S phase and a reduced number in the G2 phase of the cell cycle at 2000 µg/ml of AETC. This indicated increased DNA replication but reduced cell division in the cell, which could be a possible reason for a significantly reduced cell number at 2000 µg/ml of AETC. In line with our study, it is reported that a methanol extract of TC effectively ameliorated autoimmune arthritis in the rat model, wherein TC downregulated the pro-inflammatory cytokines, mainly IL-17 and IL-1β (Sannegowda et al., 2015).

We performed genome-wide expression profiling of naive CD4^+^ Th17 cells treated with AETC to understand the probable mechanisms of action. Our transcriptomic analysis of AETC treated Th17 cells shows the downregulation of genes and pathways key to the differentiation of Th17 cells and the pathogenesis of Th17-associated inflammatory and autoimmune diseases (Figure 3). The downregulation of key regulators of Th17 differentiation *RORC, HIF1-α, PHD2, IL-23R,* and *VEGF* with downstream inhibition of genes *HK2, GLUT1,* and *GLUT3* of the glycolytic pathway on TC treatment demonstrates the anti-inflammatory activity of TC.

A decreased IL-17 mRNA expression is generally correlated to lower IL-17 protein expression. Although our current study observed no change to a slightly higher expression of IL-17 mRNA, there was inhibition of IL-17 at the protein level. Since the current study involves a multi-component herbal drug, the rationale behind this observation requires further extensive investigation. However, the phenotypic effect on Th17 cells, reduced proliferation, and inflammation, supported by reduced expression of a cascade of genes involved in the cytokine-receptor signaling pathway, is in concordance with the reported effect of TC on autoimmune mouse model studies.

Our results show a significant reduction in the expression of FoxP3 in iTreg culture, which concord with the study wherein mice infected with *Leishmania donovani* and treated with TC extract resulted in a substantial decrease in parasite load coupled with a reduced percentage of Treg cells (Sachdeva and Kaur, 2018). IFN-γ producing Th1 cells are associated with the pathogenesis of many organ-specific autoimmune diseases (Dardalhon et al., 2008). Although single IFN-γ producing cells had no effect in the autoimmune arthritis rat model, the spleen and synovial-infiltrating cells of rats treated with TC exhibited a significant reduction in the IL-17^+^IFN-γ^+^ producing cells (Sannegowda et al., 2015). A considerable reduction in IFN-γ producing cells in Th1 polarized naive CD4^+^ T cell culture was observed in our study in the same direction. The *in vitro* model utilized in the present study consisted of a uniform naive CD4^+^ T cell culture; this could be a possible reason for observing a significant inhibitory effect on IFN-γ producing cells in our study.

The results observed with TC whole extract were further supported by an *in silico* molecular docking study, wherein the binding affinity of the TC components with Th17-associated proteins and receptor molecules suggests TC as a potential therapeutic candidate exhibiting anti-proliferative activity on polarized Th17 cells, resulting in a controlled immune response under inflammatory conditions. Some compounds, namely Apigenin-6-C-glucosyl, Pinoresinol, Acetylasimilobine, Cordifolioside-B, Secoisolariciresinol-9, Formylasimilobine, Cordifolide-A showed an efficient binding affinity for the protein molecules as well as their receptors involved in the Th17-associated immune pathways (Supplementary Table S6 in Supplementary Material). Based on the binding affinity, it can be predicted that Apigenin-6-C-glucosyl and Pinoresinol will bind and block the TGFβ/TGFβR, and Acetylasimilobine and Cordifolioside-B will bind and block IL-17/IL-17R thereby preventing the binding of the inflammatory molecules to their respective ligands, resulting in decreased inflammation. Although Pinoresinol, Apigenin-6-C-glucosyl, and Acetylasimilobine are present in a few other plants, including medicinal plants, the compound Cordifolide-A, which is exclusive to TC only, showed good binding affinity (above −5.5 kcal/mol) with IL-17, IL-17R, IL-23, and IL-23R. It is evident that the metabolite constituents of TC extract can target all possible binding domains of IL-17R protein and block them so that the T-cell-mediated hyperactive pathways are not activated, causing reduced inflammation. These findings support further investigation into individual TC secondary metabolites with high binding affinity for T cell-associated proteins and receptor molecules.

T cells are a critical immune system component, eliciting a specific immune response against a foreign particle, and they are essential in maintaining immune homeostasis. The importance of T cells in autoimmune disorders, cancer, and complex disorders such as asthma, diabetes, cardiovascular diseases, *etc.*, intensifies the use of T cells as a model system to unravel the molecular and therapeutic aspects of the associated diseases. In addition to its health-promoting and homeostasis-restoring activities, TC is clinically used for treating various complex disorders, autoimmune conditions, and cancers (Ghosh and Saha, 2012; Ministry of Ayush, Govt of India, New Delhi, 2022). Therefore, to study the multi-systematic effect of TC, we have utilized the primary T cell *in vitro* culture model system in the current study. We have used a whole extract of TC for our study due to its wide availability and clinical use. However, it would be interesting to study the active compounds of AETC individually or in combination for their activity on immune cells through novel approaches like network pharmacology (Lai et al., 2020).

In the present study, primary mouse T cells were polarized into individual T cell subsets under defined polarizing conditions. The observation of reduced frequency of IL-17 and IFN-γ secreting cells in Th17 and Th1 cell subsets, respectively, and reduced expression of FoxP3 in iTregs indicate that under stimulating conditions, TC could elicit its activity on different T cell subsets. The current study, supported by other reported immunomodulatory activity of TC on B cells, DCs, and macrophages, suggest that TC tends to restore immune homeostasis by eliciting its activity on various immune cells (Raghu et al., 2009; Aranha et al., 2012; Pandey et al., 2012; Pandey et al., 2014). However, its role in purified human T cells needs further investigation using *in vitro* model systems and clinical trials.

**FIGURE 6:**
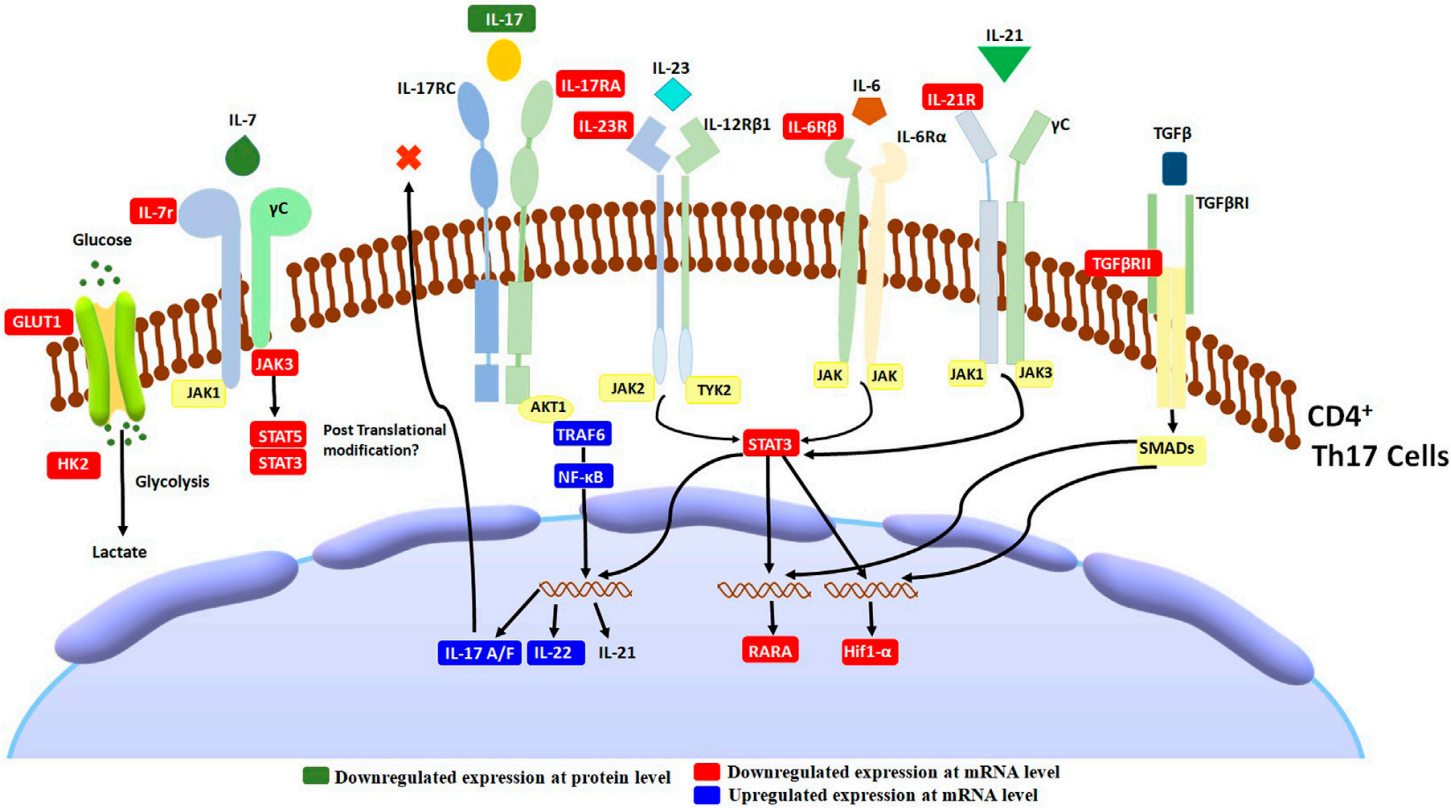
Schematic diagram of *T. cordifolia* therapeutic effects on Th17-associated inflammation. AETC treatment exerts its effect by reducing the expression of genes involved in Th17 cell differentiation, cytokine-receptor signaling, and the JAK-STAT signaling pathway.

In addition to TCR and co-stimulatory signals, T cell activation requires cytokine receptor signals (Luckheeram et al., 2012). Cytokines are essential for T cells’ development, differentiation, and function. Most of the cytokines activate the JAK-STAT pathway, which further activates the cascade of events responsible for the transcription of genes required for T cells’ development, differentiation, and function (Seif et al., 2017). In our study, an interesting observation was the downregulation of cytokine receptors, mainly belonging to the family of type 1 cytokine-receptor signaling pathways. These receptors included IL-12RB2, IL-17RA, IL-1R2, IL-21R, IL-6ST, and IL-7R. The differentiation of Th17 cells requires these receptors. In addition, TGFβR2, a receptor considered to be important for Th17 cell differentiation, was also downregulated. Altogether, from the observed cellular and gene expression level effects of AETC, supported by the molecular docking analysis, we hypothesize that AETC alters the cytokine-receptor signaling pathway involving the JAK-STAT signaling pathway in Th17 cells, along with downregulation of genes which are the key regulators in defining Th17 cells (Figure 6). Broadly, in-depth analysis at the cellular and molecular level substantiates the inhibitory effect of AETC on Th17 cells. This study was performed on Th1, Th17, and iTreg *in vitro* cell culture systems which mimics an extreme inflammatory condition wherein TC exhibited its significant inhibitory activity. Another interesting observation from our study is the downregulation of CXCR4, a receptor for HIV entry into T cells, as the second most downregulated gene in the transcriptomic profile.

In light of the above results, it can be concluded that AETC has potent immunomodulatory properties. AETC inhibited the proliferation of CD4^+^ T cells and secretion of IL-17 in the Th17 polarized cell culture system. This activity was observed *via* the cytokine-receptor signaling pathway involving the JAK-STAT signaling cascade, suggesting the potential immune therapeutic application of TC in Th17-associated inflammatory diseases. The docking results provide insight into the active ingredients involved in the immunomodulatory activity of TC on Th17 cells; however, the validation of the activity of individual components remains to be further elucidated. In addition, AETC showed inhibition of FoxP3 expression and secretion of IFN-γ in iTreg and Th1 cell subsets, respectively.

The broad spectrum activity of TC *in vitro*, *in vivo*, and clinical trial studies together suggests its immune homeostasis maintaining capacity depending upon the disease condition type. Further, a comprehensive study on other immune subsets is needed to understand and validate the biological mechanism of AETC action. This work integrates the ancient knowledge of Ayurveda with the modern tools of science, resulting in deciphering the multi-dimensional activity of the herb TC on T cells.

## Data Availability

The datasets presented in this study can be found in online repositories. The names of the repository/repositories and accession number(s) can be found below: NCBI Gene Expression Omnibus [GEO] (https://www.ncbi.nlm.nih.gov/geo/), GSE201104

## Abbreviations

AETC, Aqueous extract of *Tinospora cordifolia*; EMA, ethidium monoazide; GSEA, Gene set enrichment analysis; IFN-γ, interferon-γ; PI, propidium iodide; RR1, (1,4)-α-D-glucan (RR1); TC, *Tinospora cordifolia*; TGFβ, transforming growth factor-beta; Treg, T regulatory.
