# Supplementary material for: Deciphering the mechanism of Tinospora cordifolia extract on Th17 cells through in-depth transcriptomic profiling and in silico analysis
Source: Front Pharmacol. 2023 Jan 9;13:1056677. doi: 10.3389/fphar.2022.1056677 (PMC9868420; doi:10.3389/fphar.2022.1056677)
Supplement: Supplementary file 7 [file DataSheet1.docx]

File_1

Compounds reticuline (Rt 10.62) m/z 330 [M+H]+, tinocordiside (Rt 8.14) m/z 419 [M+Na]+, 20β-Hydroxyecdysone (Rt 10.83) m/z 481 [M+H]+, cordifolioside A (Rt 5.82) m/z 503 [M+Na]+, tinosporinone (Rt 5.24) m/z 342 [M]+, 1,2-dihydroxyaporphine (R)-form, O1-Me, N-de-Me, N-formyl, 2-O-[β-D-glucopyranosyl-(1→2)-β-D-glucopyranoside] (Rt 7.86) m/z 601 [M+H2O]+, jatrorrhizine (Rt 10.2.) m/z 338 [M]+, cordifolide A (Rt 8.48) m/z 621 [M+Na]+ were identified on the basis of LC-MS results.

**Reticuline (R_t_ 10.62) *m/z* 330 [M+H]^+^**

**Tinocordiside (R_t_ 8.14) *m/z* 419 [419+Na]^+^**

**20β-Hydroxyecdysone (R_t_ 10.83) *m/z* 481 [M+H]^+^**

**Cordifolioside A (R_t_ 5.82) *m/z* 503 [503+Na]^+^**

**Tinosporinone (R_t_ 5.24) *m/z* 342 [M]^+^**

**Dihydroxyaporphine; (*R*)-form, *O*^1^-Me, *N*-de-Me, *N*-formyl, 2-*O*-[β-D-glucopyranosyl-(1→2)-β-D-glucopyranoside] (R_t_ 7.86) *m/z* 601 [M]^+^**

**Jatrorrhizine (R_t_ 10.2) *m/z* 338 2[M-1]^-^**

**Cordifolide A (R_t_ 8.48) *m/z* 621 [621+Na]^+^**

^^
